# CropDeep: The Crop Vision Dataset for Deep-Learning-Based Classification and Detection in Precision Agriculture

**DOI:** 10.3390/s19051058

**Published:** 2019-03-01

**Authors:** Yang-Yang Zheng, Jian-Lei Kong, Xue-Bo Jin, Xiao-Yi Wang, Ting-Li Su, Min Zuo

**Affiliations:** 1 School of Computer and Information Engineering, Beijing Technology and Business University, Beijing 100048, China; zhengyangyang@st.btbu.edu.cn (Y.-Y.Z.); jinxuebo@btbu.edu.cn (X.-B.J.); sdwangxy@163.com (X.-Y.W.); sutingli@btbu.edu.cn (T.-L.S.); zuomin@btbu.edu.cn (M.Z.); 2 Beijing Key Laboratory of Big Data Technology for Food Safety, Beijing Technology and Business University, Beijing 100048, China

**Keywords:** Internet of Things, agricultural autonomous robots, deep convolutional neural networks, real-time online processing, greenhouse

## Abstract

Intelligence has been considered as the major challenge in promoting economic potential and production efficiency of precision agriculture. In order to apply advanced deep-learning technology to complete various agricultural tasks in online and offline ways, a large number of crop vision datasets with domain-specific annotation are urgently needed. To encourage further progress in challenging realistic agricultural conditions, we present the CropDeep species classification and detection dataset, consisting of 31,147 images with over 49,000 annotated instances from 31 different classes. In contrast to existing vision datasets, images were collected with different cameras and equipment in greenhouses, captured in a wide variety of situations. It features visually similar species and periodic changes with more representative annotations, which have supported a stronger benchmark for deep-learning-based classification and detection. To further verify the application prospect, we provide extensive baseline experiments using state-of-the-art deep-learning classification and detection models. Results show that current deep-learning-based methods achieve well performance in classification accuracy over 99%. While current deep-learning methods achieve only 92% detection accuracy, illustrating the difficulty of the dataset and improvement room of state-of-the-art deep-learning models when applied to crops production and management. Specifically, we suggest that the YOLOv3 network has good potential application in agricultural detection tasks.

## 1. Introduction

Modern agriculture seeks to manage crops in controlled environments such as greenhouses, that are able to improve the production of plants or duplicate the environmental conditions of specific geographical areas to obtain imported products locally. Moreover, severe weather and diseases variations that impact on crop production and quality can be avoided with a comprehensive application of new monitoring and information technologies including Internet of Things (IoT), autonomous robots and smartphones [1]. Now it is possible to obtain highly accurate status of crops and form reasonable decisions to manage irrigation, change climate factors, or enrich the soil nutrition in agricultural scenes, which optimize automation of precise management and improve production of crops while potentially reducing environmental impacts [2]. Farmers and agronomists have already begun employing technologies in order to improve the efficiency of their work in greenhouses [3]. With the sensor data obtained and transmitted by IoT, they use smartphones to remotely monitor their crops and equipment, understand the whole management status accurately with statistical analysis, and instruct the robots to carry out agricultural tasks. Although the greenhouses are taking advantage the integration of different technologies with efficient human intervention, the current level of artificial intelligence (AI) in agricultural machines and systems is far from achieving automated operations and management requiring minimum supervision to optimize production by accounting for variability and uncertainties within precision agriculture (PA) [4]. 

Intelligence has been considered as the further enabler and the important technological challenge in promoting economic potential and ecological value of the whole PA. To this end, the development of deep learning technology has provided an effective approach to facilitating intelligent management and decision-making in many aspects of PA, such as visual crop categorization [5], real-time plant disease and pest recognition [6], picking and harvesting automatic robots [7], healthy and quality monitoring of crop growing [8]. Moreover, there is increasing agricultural success present in the near future because deep-learning systems can easily take advantage of data increases in the number of available sensors, cameras, and smartphones. Inspired by the multi-level visual perception process of human brain, deep-learning allows computational models that are composed of multiple processing layers to learn representations of data with multiple levels of abstraction, obtained by non-linear modules (such as convolutional layers or memory units) that each transform the representation at one level (starting with the raw input) into a representation at a higher, slightly more abstract level [9]. With the composition of enough such transformations, very complex functions can be learned and tough structures in high-dimensional data can be discovered automatically to complete agricultural tasks.

However, deep-learning networks achieving state-of-the-art performance in other research fields are not suitable for agricultural tasks of crops management such as irrigation [10], picking [11], pesticide spraying [12], and fertilization [13]. The dominating cause is that there are no public benchmark datasets specifically designed for various agricultural missions, which limits the further application of deep-learning technology and the wider development of intelligence in greenhouses. These situations demonstrate the need to construct appropriate crop datasets by taking full advantage of various collection devices for deeper and wider networks to generate better results.

To assist the identification and detection of different crops that characterize the agricultural missions, we introduce a novel domain-specific dataset named CropDeep, which consists of vegetables and fruits that are closely associated with PA. Currently, the dataset covers common vegetables and fruits of 30 categories, which are collected by visual cameras of IoT, autonomous robots, and smartphones in greenhouses. It contains more than 31,000 images in total and at least 1500 samples for each class with well annotation, which are suitable for the subsequent categorization and detection of agricultural tasks. Furthermore, CropDeep not only has different parts and different growth periods of a certain vegetables or fruits, but also similar parts of different species, such as leaves and flowers, are classified into separate classes, which present the domain-specific complexity in realistic PA compared to other public datasets sourced from social media or the Internet. To characterize the classification and detection difficulty of CropDeep, we also ran experiments with several state-of-the-art deep-learning frameworks with utilizing the different species in real scenarios. By comparing the performance of different classification and detection models, confirming the classification and detection architectures with faster speed and high accuracy is the main purpose of this study, which verifies that the effective application of CropDeep is well-suited for the sensors and equipment in PA, providing accurate scientific data for agricultural production and management of crops. 

The rest of the paper is organized as follows. Section 2 describes the related dataset to this study; Section 3 presents a specific description of CropDeep datasets in aspects of data collection, construction, annotation, and division. Section 4 introduces the deep-learning classification and detection network selected. Section 5 presents experimental results and discussion of the dataset to verify the application performance. Finally, we present our conclusion with further research aims in Section 6.

## 2. Related Datasets

In this section, we will review the image classification test datasets of existing plants or crops commonly used in computer vision. We focus on large-scale, annotated crop object detection, rather than datasets with common everyday objects. In Table 1, we summarize the statistics of some of the most common datasets for plants or crops.

We analyzed the datasets in Table 1. These datasets are broadly divided into two categories, one related to agricultural production. These datasets contain all the plants, but the amount of data is relatively small. Most of the datasets are only used for classification tasks, and there is no annotation for the detection tasks. Data sources are basically collected by laboratory cameras, such as the Flowers 102 [14], LeafSnap [15], PlantVillage, and Urban Trees datasets [16].

Flowers 102 consists of 102 flower categories. The flowers chosen are flowers commonly occurring in the United Kingdom. Each class consists of between 40 and 258 images. The images have large scale, pose, and light variations. In addition, there are categories that have large variations within the category and several very similar categories. The dataset is visualized using isomap with shape and color features.

The Plant Village Dataset contains only images of leaves that are previously cropped in the field and captured by a camera in the laboratory. This is unlike the images in our Tomato Diseases and Pest Dataset, which are directly taken in-place by different cameras with various resolutions, including not only leaves infected by specific pathogens at different infection stages but also other infected parts of the plant, such as fruits and stems. Furthermore, the challenging part of our dataset is to deal with background variations mainly caused by the surrounding areas or the place itself (greenhouse).

The other type of dataset is mainly a general-purpose database with a large amount of data, mainly used for competitions. These datasets have many categories, and each category contains a large number of category samples, which is the main object of the current deep learning model. Although these datasets are all public, their image sources are mainly web crawling, which is not suitable for agricultural production, such as ILSVRC2012 [17], Microsoft coco [18], CUB 200-2011 [19], and iNat2017 datasets [20]. Although these databases have classification and detection tasks, high-quality images with large data volumes, and high-quality annotations, the proportion of plants, especially crops, in these databases is particularly small.

The ImageNet dataset is currently the world’s largest collection of image classification data, containing 14 million images, 22,000 types, and an average of 1000 images per type. In addition, ImageNet has built a dataset containing 1000 objects, with 1.2 million images, and used this dataset as a data platform for image recognition competitions.

The MS coco dataset contains more than 300,000 images, more than 2 million annotation objects, and 80 object types. Although there are fewer types than ImageNet and SUN, there are many images for each type of object, which is the current dataset with the largest number of targets per image. MS COCO can be used not only for target detection research, but also for studying contextual relationships between objects in an image.

Unlike web scraped datasets [21], the annotations in iNat2017 represent the consensus of informed enthusiasts. Images of natural species tend to be challenging as individuals from the same species can differ in appearance due to sex and age, and may also appear in different environments. Depending on the particular species, they can also be very challenging to photograph in the wild. In contrast, mass-produced, man-made object categories are typically identical up to nuisance factors, i.e., they only differ in terms of pose, lighting, color, but not necessarily in their underlying object shape or appearance.

In recent years, there have been continuous developments in agriculture, such as AI Challenge and VegFru [22]. The task of these competitions is to classify plants without the task of detection. Therefore, the dataset of the game does not have an annotation about the detection, so the dataset cannot be used for the detection task. VegFru is a new domain-specific dataset for fine-grained visual classification (FGVC) [23]. VegFru is a larger dataset consisting of vegetables and fruits which are closely associated with the daily life of everyone. Aiming at domestic cooking and food management, VegFru categorizes vegetables and fruits according to their eating characteristics, and each image contains at least one edible part of vegetables or fruits with the same cooking usage. Particularly, all the images are labelled hierarchically. The current version covers vegetables and fruits of 25 upper-level categories and 292 subordinate classes. It contains more than 160,000 images in total and at least 200 images for each subordinate class.

This paper presents the CropDeep Agricultural Dataset due to the lack of a crop database for testing tasks. All images of CropDeep are collected by various equipment including cameras of IoT, autonomous spray robot, autonomous pinking robot, mobile cameras, and smartphones in an intelligent agricultural monitoring and management platform. Using these sensors to collect images can be better applied to the agricultural environment, making detection and classification tasks more realistic. CropDeep marks a large number of crop images for collection, and each category contains a large number of samples, which is suitable for precision agriculture. The most distinctive feature of CropDeep dataset images is the different growth seasons that contain certain vegetables or fruits that are not included in many other datasets. The small gaps between categories of some classes make it difficult to classify and detect fruits and vegetables in the greenhouse. Therefore, the CropDeep dataset with fine-grained features [24] is challenging for accurate detection of training deep learning models.

**Figure 1:**
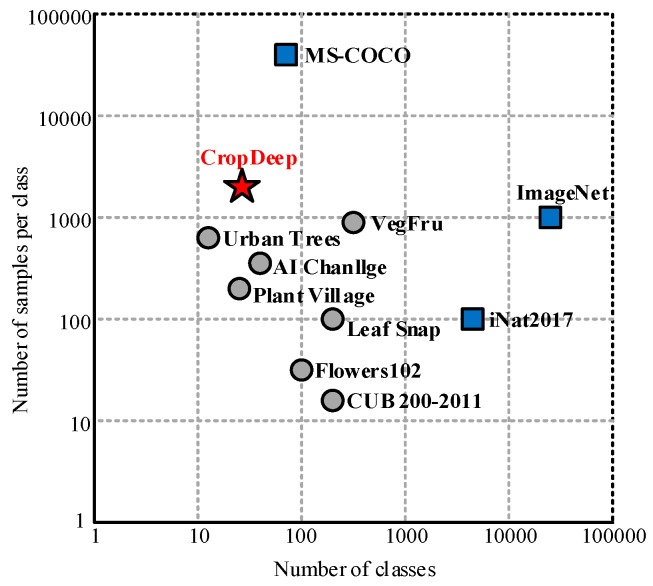
Basic dataset comparison chart.

The comparison will be based on the number of images included in the dataset, the number of types, and the number of samples per category. An intuitive comparison is shown in Figure 1. The circle represents the plant dataset, the square represents the non-plant dataset, the gray represents the dataset containing only the classification, and the blue represents the dataset containing the classification and detection.

## 3. CropDeep Overview

In this section, we describe the details of the CropDeep dataset, including how we collected the image data in greenhouses (Section 3.1), how we constructed the species of crops (Section 3.2), and how we annotated bounding boxes and defined train, validation, and test splits (Section 3.3). The overall experience of the dataset is introduced to future researchers for constructing their own datasets.

### 3.1. Dataset Collection

**Figure 2:**
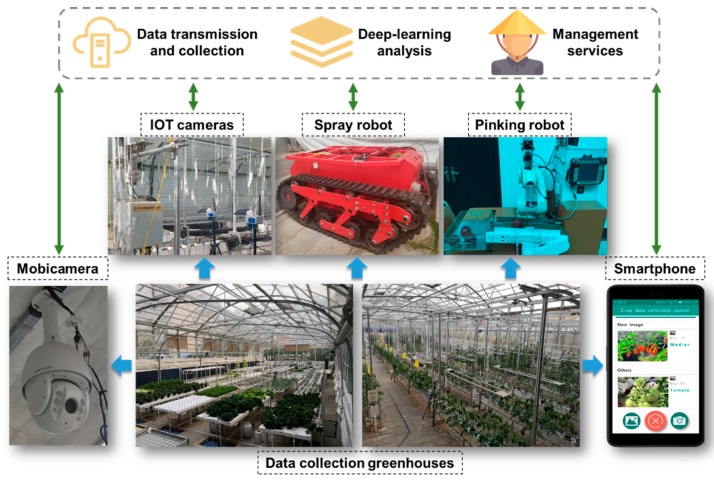
The dataset collecting process of agricultural monitoring and management platform.

Unlike other web scraped datasets, all images of CropDeep are collected by various equipment including cameras of IoT, autonomous spray robot, autonomous pinking robot, mobile cameras and smartphones in an intelligent agricultural monitoring and management platform, which aims at providing deep-learning analysis and management services for better crops production as presented in Figure 2.

Each device takes pictures of crops and uploads them to the cloud server in CAN or wireless fidelity way for different purposes. For example, cameras of IoT and mobile cameras are used to collect information about the entire farmland area in order to monitoring the detailed growing status of specific crops. The IoT cameras collect photos of the growth process of each crop in the greenhouse, where it captures the growth of crops at different times. Pictures taken by IoT cameras generally contain only one type of crop, which are set as a training set. The photos taken by the mobile camera contain the growth status of a variety of crops, and we make the pictures taken as a test set. The images taken by IoT cameras and mobile cameras are not the same size. We cut the images to 1000 × 1000 px size. Additional data from other measurements can also flow into this data, such as biomass distribution and weather data. Those collected data are submitted to computers in the platform and combined with plant cultivation rules and regulations in order to determine management measures. The offline decision processes based on static data permit an appropriate time delay on transferring resulting instructions. In contrast, agricultural machines and robots independently collect images of crops and immediately convert them into management measures, allowing for a high level of spatial and seasonal dynamic. Those field maintenance tasks are severely dependent on the real-time performance of online decision-making algorithms and stored in the platform. Subsequently, the smartphones collected the crops images in order to meet more complex tasks of farmers and agronomists, which do not only allow to continuously monitor the health and growth of the cows, but also to determine the operation measures for autonomous robots. Moreover, the images of smartphones play a significant role in social contact and sharing. Therefore, it is very challenging to classify and detect crop species from massive images with different angles, focal lengths, and resolutions offered by various devices in the platform. 

To meet the precision and efficiency of various aspects, the CropDeep datasets are specially designed to carry out agricultural tasks of PA management in greenhouses. Depending on data integration of the devices, CropDeep offer the abundant attributes of particular crops and environmental factors underlying object shape or appearance in the greenhouses, where the tables of crops are arranged in rows. In contrast, other web-collected datasets are typically identical up to nuisance factor and only differ in terms of pose, lighting, color. In the long run, the effective and useful implementation of CropDeep will promote the nationwide development of PA in the future, especially in rural areas.

### 3.2. Dataset Construction 

On the basis of various devices and equipment, the CropDeep dataset has collected 31,147 images including vegetables, fruits, and people in laboratorial greenhouses. The sizes of all images were cropped and resized to less than the maximum 1000 × 1000 px, which are limited by the standard input of existing deep-learning detection network. Currently, the dataset covers vegetables and fruits of 19 upper-level categories and 30 subordinate classes, which are the most reasonable for the PA purpose and arranges crops into root vegetable, cabbage, leafy vegetable, melon fruits, etc. Therefore, the dataset is divided into the following 31 categories according to the agricultural biological taxonomy: four growth stages of tomato (*Lycopersicon esculentum Mill.*) including ripe, unripe, early-blossom and full-blossom; three growth stages of cucumber *(Cucumis Linn.*) including ripe, unripe, and blossom; four species of lettuce (*Lactuca sativa Linn.*) including head lettuce, butter lettuce, luosheng lettuce, and iceberg lettuce; two species of cabbages (*brassica oleracea linn.*) including purple cabbage and Chinese cabbage; and two species of turnips (*raphanus sativus linn.*) including ternip and green turnip. In addition, the dataset contains five leafy vegetables including endive (*Picris divaricata Vaniot.*), rutabaga (*Brassica napobrassica Mill.*), celery (*Apium graveliens Linn.*), spinach (*Spinacia oleracea Linn.*), scallion (*Allium fistulosum Linn.*); and four frugivorous vegetables including fingered citron (*Sechium edule Jacq.*), winter squash (*Cucurbita maxima Duch.*), pumpkin (*Cucurbita moschata Duch.*), chili pepper (*Capsicum annuum Linn.*). The other species are various fruits including lemon (*Citrus limon Burm.*), persimmon (*Diospyros kaki Thunb.*), pawpaw (*Chaenomeles Lindl.*), watermelon (*Citrullus lanatus Matsum.*), and muskmelon (*Cucumis melo Linn.*). Moreover, a unique medicinal fruit wolfberry (*Lycium barbarum Linn.*) and persons working in greenhouses are listed in CropDeep dataset. The construction principles guide the process of image collection, as well as the detail quantity of images and annotated samples listed in Table 2, which is a really challenging project.

**Figure 3:**
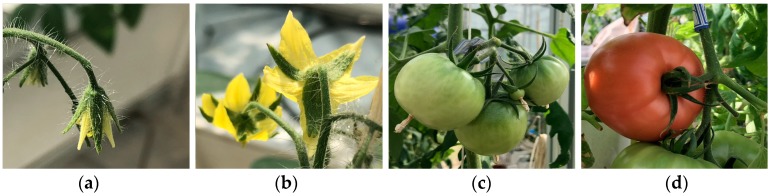
Different growth period states of tomato. Left to right: (**a**) early-blossom, (**b**) full-blossom, (**c**) unripe, and (**d**) ripe.

The most special characteristic of images in CropDeep contain different growth periods for a certain vegetable or fruit, which is not included in lots of other datasets, as shown in Figure 3. Besides, some crops categories are firstly covered in detecting application based on deep-learning methods such as wolfberry and luosheng lettuce. This means that CropDeep datasets and subsequent deep-learning models can monitor the growth and healthy status of fruits and vegetables and make better decisions to improve the PA management in greenhouses.

**Figure 4:**
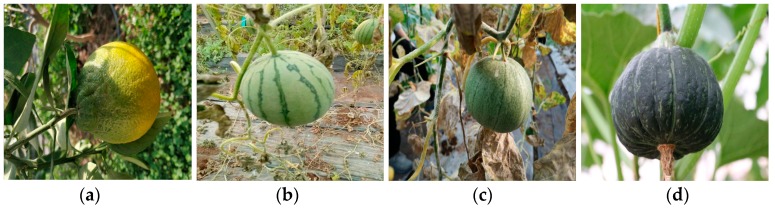
These images belong to different classes but with subtle interclass difference. Left to Right: (**a**) winter squash, (**b**) watermelon, (**c**) muskmelon, and (**d**) pumpkin.

Compared to these existing datasets, the domain of CropDeep datasets is novel and more associated with agricultural tasks and people’s daily life, which contributes to its broad application prospects. CropDeep is larger in scale, which has up to 1100 annotated samples available in each class at least for deep-learning model training. Particularly, some species are hierarchically assigned with fine-grained labels, which is well-suited for the hybrid granularity research of different devices in PA management. However, the subtle gaps among some inter-class categories make it difficult to classify and detect fruits and vegetables in greenhouses as shown in Figure 4. Therefore, CropDeep datasets with the fine-grained characteristics are challenging to train deep-learning models for precise detection, which is worth investing future research.

### 3.3. Data Annotation and Division 

**Figure 5:**
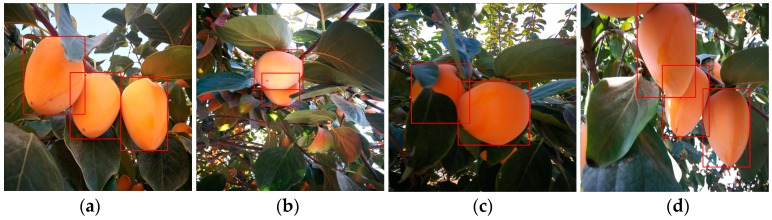
Annotating principle of CropDeep dataset.

The next step was annotation process, which labels locates a bounding box on a crop in the image and export the corresponding class and location information. At this point, it is necessary to illustrate the differences between the notions of image classification and object detection. Classification estimates if an image contains any instances of an object class, while detection approach deals with the class and location instances of any particular object in the image. Detection is more complicated than classification, as well as the number of samples for detection is also more than images number for classification. In practical agricultural missions, a single image usually contains multiple objects of various categories, which should be estimated with the class probability of crop and its location. In this study, the annotating process of CropDeep is labor-intensive and similar to PASCAL Visual Object Classes dataset [25]. The labeling principle of datasets has the following guidelines:

(1) When a picture contains multiple objects, each instance should be marked out (e.g., Figure 5a).
(2) When there are two overlapped instances in the picture, the occluded parts should be draw with the box around the visible parts. The instances should be completely enclosed and marked (e.g., Figure 5b).
(3) When there are other blurred instances in the background, if it is extremely small and difficult for people to distinguish, ignore it. If it is easy to distinguish from the requested class—regardless of size, blurriness, or occlusion—put a box around the instance (e.g., Figure 5c).
(4) If images with multiple instances of the super-class, all of them are boxed, up to a limit of 5, even bounding boxes may overlap. If the instances are physically connected, then only one box is placed around them (e.g., Figure 5d).

Under the above guidelines, 49,765 bounding boxes were obtained from 31,147 images in the datasets. The annotated percentage of all crops centralized distribution in the interval of 2.5% and 3.9% except for person, which illustrate that the dataset has a good balance in avoids learning differences of detecting models to various crop species.

On the basis of fine annotation, it is time to partition the images into the train, validation, and test splits according to the deep-learning methods. For each of selected species, we divide the datasets into training sets, validation sets, and test sets. Allowing for the samples number of each class will be different, we take 80% of each species as a training set, 10% for the validation set, and 10% for the test set. These empirical proportions make up the imbalance problem in the dataset. Even if there are some classes with a larger number of training samples, their corresponding test sets also contain more samples, suffering more rigorous assessment. At this point, we have the final image splits, with a total of 24,917 training images, 3120 validation images, and 3110 test images. The bounding box of majority instances are relatively medium sized within a certain balance. The CropDeep dataset is available from the corresponding author by email.

## 4. Deep-Learning Classification and Detection Models

**Figure 6:**
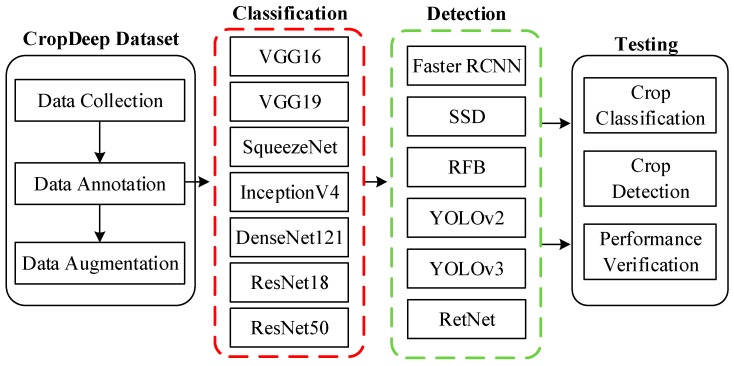
Experimental frameworks of deep-learning detection and classification model for various crops in the CropDeep dataset.

With the development of deep learning and high-computational hardware technology, deep-learning-based model—especially convolutional neural networks (CNN)—for classification and detection have been increasingly proposed, showing good performance on different crops [26]. However, those models offered management services on the basis of crops images captured in the laboratory or crawled from Internet, which is far from seeing agricultural application in PA. This is unlike the images in our CropDeep dataset, which are directly taken in-place by different devices and equipment in greenhouses. Although the state-of-the-art deep-learning frameworks show outstanding performance on crops recognition, the challenges dealing with agricultural tasks are still difficult to overcome. Therefore, to characterize the classification and detection difficulty of CropDeep, we ran experiments with several state-of-the-art deep-learning classifiers and detectors. Our task is to consider a technique that not only recognizes the crop species in the image but also to identify its location for the posterior development of real-time agricultural management. An overview of the experimental frameworks of deep-learning classification and detection is shown in Figure 6.

### 4.1. Deep-Learning Classification Models 

As mentioned in the previous section, deep convolutional neural networks have become the dominating approach for image classification. Year after year, various new architectures have been proposed. When choosing a deep architecture to solve a realistic problem, some aspects should be taken into consideration such as the type or number of layers, as a higher number of parameters increases the complexity of the system and directly influences the memory computation, speed, and results of the system. Although designed with specific characteristics according to realistic applications, the current deep-learning network has the same goal to increase accuracy while reducing operation time and computational complexity. Therefore, this study selected some state-of-the-art deep-learning architectures—including VGG [27], ResNet [28], DenseNet [29], Inception [30], and SqueezeNet [31]—to assess the performance on the CropDeep dataset and the outstanding applicability for our management platform.

VGG-16 was proposed by the famous VGG (Visual Geometry Group) of Oxford University. Due to its good generalization performance, VGG-16 can improve the classification accuracy by using its pre-trained model on the ImageNet dataset. Small convolution kernels (3 × 3) are commonly used in VGG-16 to increase network depth for better capacity. Another typical extension model is VGG-19 with adding fully-connected layers and pooling layers. Both of VGG-16 and VGG-19 required input data with unified dimensions of 224 × 224 × 3, and were selected to identify various crops.

Since deeper neural networks are more difficult to train, a residual learning framework is proposed to mitigate network training with deeper structure, which was defined as ResNet. This model explicitly reformulated the layers as learning residual functions with reference to the layer inputs instead of learning unreferenced functions. Partial data of the input goes directly to the output without passing through the neural network. This was proven to preserve some original information and prevent effectively the gradient dispersion problem in back propagation. Similarly, the basic idea of DenseNet was derived from ResNet, but it established the dense connection between all the previous layers and the latter layers. Another dominating characteristic of DenseNet was reusing feature maps of each layers through the connection of the channel. DenseNet was to alleviate the vanishing gradient problem, strengthen feature propagation, encourage feature reuse, and substantially reduce the number of parameters.

As the increasing of depth promotes the classifying ability of the deep-learning network, the width also has similar performance. Inception is to increase in breadth by using different convolution kernel sizes, 3 × 3, 5 × 5, and 1 × 1. These different convolution kernels extract different features and increase the diversity of features. However, this caused a problem that the parameter quantity increases too much. In order to solve this problem, a 1 × 1 convolution kernel dimension reduction is introduced to make a balance between computational efficiency and parameter quantities. With equivalent accuracy, smaller CNN architectures offer at least three advantages: (1) Smaller CNNs require less communication across servers during distributed training. (2) Smaller CNNs require less bandwidth to export a new model from the cloud to an autonomous car. (3) Smaller CNNs are more feasible to deploy on FPGAs and other hardware with limited memory. Therefore, the SqueezeNet model was released with fewer parameters and high accuracy while broadly exploring the design space of CNN architectures.

In this study, we hope that those deep-learning classification models will explore the broad range of possibilities in the application of CropDeep dataset and to perform that exploration in a more compiled systematic manner.

### 4.2. Deep-Learning Detection Models 

Convolutional neural networks are presently considered the leading method for object detection. Currently, the deep-learning detection models were mainly divided into two-stage object detectors and single-stage object detectors. For two-stage detection network, a sparse set of candidate object boxes is first generated, and then they are further classified and regressed. The basic region-based convolutional neural network (R-CNN) [32] is the earliest application of CNN features to construct the detection system with well performance. Then a fast region-based convolutional neural network (Fast R-CNN) [33] is proposed to combine the target classification with bounding box regression to solve multi-task detection. Furthermore, the regional proposal network (RPN) is proposed by the faster region-based network (Faster R-CNN) [34] to generate amounts of anchors in two stages, which are richer proposals to improve accuracy slightly. In the first stage, a RPN takes an image as input and processes it by a feature extractor based on intersection-over-union (IoU) [35] between the object proposals and the ground-truth. In the second stage, the box proposals previously were generated to crop features from the same feature map to predict the class probability and bounding box of each region proposal. While Faster R-CNN has been a milestone of the two-stage detectors, these algorithms have some disadvantages in dealing with resources and time consumption for large datasets, which is not suitable for subsequent realistic application of object detection.

Therefore, single-stage object detection networks with faster speed are trained by regular and dense sampling over locations, scales, and aspect ratios in an end-to-end flow. The main advantage of single-stage method is its high improvement in computational efficiency greatly, which is suitable for realistic tasks. The representative methods such as ‘You only look once’ v2 (YOLOv2) [36] simplify object detection as a regression problem, which directly predicts the bounding box and associate class probabilities without proposals generation. However, the single-stage detection accuracy is lower than that of the two-stage, as long as it is caused by a class imbalance problem. Some recent methods in the one-stage approach aim to address this dilemma, to improve the detection accuracy. Single shot multibox detector (SSD) [37] improves accuracy performance by producing different scale predictions and fusing feature maps of different layers. Similarly, receptive field block network (RFB Net) [38], inspired by the receptive fields of human vision, proposes a novel RFB block module to significantly reduce the search space of objects. Combining the characteristics of the above models, YOLOv3 [39] design a feature extractor with 53 convolutional layers (Darknet-53) to improve YOLOv2 in several aspects. Then feature pyramid networks and binary cross entropy loss were merged for class prediction in training. Furthermore, RetNet [40] applied focus loss and bounding box regressor to reduce false positives resulting from class imbalance, which has been one of the best detectors with excellent performance in accuracy, speed, and complexity trade-offs. 

In our opinion, the current two-stage and single-stage methods build the framework tone of the target detection together. The essential difference between two detectors is the tradeoff between the recall and localization, which fundamentally determine the accuracy and detection time. The single-stage detector has a higher recall at cost of low localization. Instead, the two-stage detector has a higher positioning capability, but the recall is lower, since the refine of the box’s precision could kill some positive samples by mistake. Both of above methods have achieved top performances on several challenging benchmark. However, the detection effect is still unclear in face of special inspection tasks on our dataset. Therefore, we ran some state-of-the-art models including Faster R-CNN, SSD, RFB Net, YOLOv2, YOLOv3, and RetNet to test the performance of crop detection in our CropDeep dataset. The detail results of each classification and detection model in compared experiments are presented in following section.

## 5. Experiments

In this section, we compare the performance of state-of-the-art deep-learning models on CropDeep datasets. The results are presented in classification and detection separately. 

### 5.1. Classification Results

To avoid over-fitting of the network, some augmented preprocessing was applied to enhance a larger amount of images in the dataset before training. These augmentations consist of geometrical transformations (resizing, random crop, rotation and horizontal flipping, aspect ratio) and intensity transformations (contrast and brightness enhancement, color, noise). Resizing is the adjustment of the size of the image input to the network. When training the network, the input picture size is diversified, so that the network has better generalization. Random crop is a random sample of the original image. We resize the randomly cropped portion to the size of the original image and enter it into the network. Rotation rotates the image randomly by 0–360 degrees, increasing the generalization of the network. Horizontal flipping is to flip the image 180 degrees horizontally in order to increase the diversity of the image. Contrast is in the HSV color space of the image, changing the saturation S and V luminance components, keeping the hue H constant, exponentially calculating the S and V components of each pixel (exponential factor between 0.25–4), increasing the illumination variation. Color is to add random perturbations on the image channel. Noise is a random perturbation of each pixel of the image, and the commonly used noise modes are salt and pepper noise and Gaussian noise. Moreover, we consider another augmentation method called mixup [41]. In the mixup training, each time a new example was formed by a weighted linear interpolation of two random examples. Then, the mix parameter was set to 0.2 and increase the number of epochs from 120 to 200 because the mixed examples ask for a longer training progress to converge better. 

As the datasets are divided into 80% training sets, 10% verification sets, and 10% testing sets, the experiments with seven deep-learning classification architectures—including VGG16, VGG19, Squeezenet, InceptionV4, Densenet121, Resnet18, and Resnet50—were carried out. All models were trained and tested on an Intel Core i7 3.6 GHz processor with four NVIDIA Tesla p40 GPUs and 256 G RAM. The training is proceeded on the training set, after that the evaluation is performed on the validation set for minimizing overfitting. When the training process and parameter selection were achieved, the final evaluation is done on the unknown testing set for evaluating the performance. Training batches of size 64 were created by uniformly sampling from all available training images as opposed to sampling uniformly from the classes. We fine-tuned all networks from ImageNet pretrained weights with a learning rate of 0.0001, decayed exponentially by 0.94 every four epochs, and RMSProp optimization with momentum and decay both set to 0.9. Training and testing were performed with an image not exceeding 300 px in size, with a single centered crop at test time.

#### 5.1.1. Performance Comparison

Table 3 summarizes the accuracy performance of the models. ‘Average accuracy’ indicates the average number of training images per class for each class. We observe a large difference in performance across the different crops. From the ResNet family, the higher capacity ResNet50 outperforms the ResNet18 network. As a comparison, Densenet121 is comparable to ResNet18. VGG16 and VGG19 performed worse on CropDeep datasets compared to the above architectures, likely due to over-fitting on categories with small number of training images. Similarly, the addition of the Inception reduced the performance for both fruits and vegetables comparing to the VGG architectures due to the small amount of crop species. SqueezeNet, designed to efficiently run on embedded devices, had the lowest performance. 

As illustrated in Table 3, the ResNet50 was the best performing model with achieving an average accuracy of 99.81% on the CropDeep datasets. With the accuracy of DenseNet121 and ResNet18 over 99%, an application rule of our dataset is obtained that the deeper network framework, the higher classification accuracy, but the performance will reduce as layers of framework over 50. In contrast, the accuracy of Inceptionv4 only reached 96.89%, which is 2.9% lower than ResNet50. SqueezeNet is a lightweight network with an accuracy of only 94.08%. However, it produces fewer parameters with a higher operating speed. Two VGG networks are relatively stable achieving over 98.5% without too many parameters. Overall, the CropDeep dataset is finely suitable for various deep-learning classification model, which provides an effective data foundation for various agricultural tasks.

#### 5.1.2. Loss Function

**Figure 7:**
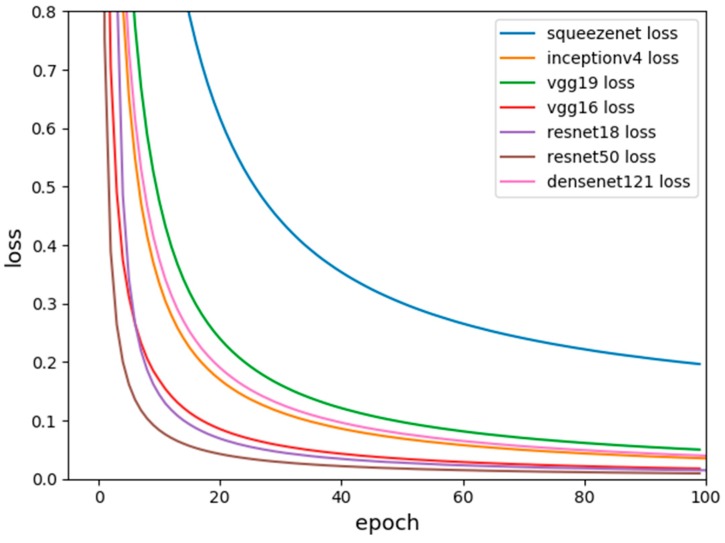
Loss function graphs of each classification network.

The loss function is used to estimate the degree of inconsistency between the predicted value of models and the true value. It is a non-negative real-valued function, usually represented by L1 or L2 regularization term. The loss function used by our network is the cross-entropy loss function. The formula for the loss function is

(1) $$J=-\sum_{c=1}^{M}y_{c}\log(p_{c})$$

where *M* indicates the number of categories. *y* indicates the indicator variable (0 or 1) if the category and sample have the same category, otherwise 0. *p* denotes the predicted probability that the observed sample belongs to category *c*. The loss function is used to estimate the degree of inconsistency between the predicted value f(*x*) of your model and the true value Y. It is a non-negative real-valued function, usually expressed by L (Y, f(*x*)). The smaller the loss function, the better the robustness of the model. The loss function is the core part of the empirical risk function. Figure 7 shows the loss function diagram for the seven models. 

The comparison of the loss function shows that the trend of the loss function values of the seven classification networks is decreasing in our dataset. It is stable for about 90 generations and the predicted value is closer to the true value. Between them, the loss function of VGG16, ResNet18, and ResNet50 drops the fastest, and the final loss function drops to about 0.0084. The loss functions of VGG19, Inceptionv4 and DenseNet are slower, and the loss function drops to around 0.0186. However, Squeezenet is a lightweight classification network with low accuracy, of which the parameters are small and the speed is fast. The relative loss function drops the slowest and eventually approaches 0.1956, which is much larger than the series of ResNet.

### 5.2. Detection Results

Along with reporting the classification accuracy, we also analyze the detection performance on each crop. Here, state-of-the art deep-learning-based detectors—including Faster-RCNN, SSD, RFB, YOLOv2, YOLOv3, and RetNet—are taken to illustrate the proof and evaluated on CropDeep dataset. On the basis of data augmentation, each model is trained with asynchronously optimized to expedite experiments on 4 GPUs. In this experiment, we train our network on the union of training set and verification set. We set the batch size at 64 and the initial learning rate of 10^−3^ as in the original model, but it makes the training process not so stable as the loss drastically fluctuates. Instead, we use a ‘warmup’ strategy that gradually ramps up the learning rate from 10^−6^ to 4 × 10^−3^ at the first five epochs. After the ‘warmup’ phase, it goes back to the original learning rate schedule divided by 10 at 150 and 200 epochs. The total number of training epochs is 500. We utilize a weight decay of 0.0005 and a momentum of 0.9. The training objective is to reduce the losses between the ground-truth and estimated results, as well as to reduce the presence of false positives in the final results, by non-maximum suppression (NMS) of each meta-architecture, which selects only candidates only with an IoU > 0.5 compared to their initial annotated ground-truth. Additionally, we evaluate the models by integrating images with a single instance and images with various species’ presence and multiple instances for promote the robustness of each model.

#### 5.2.1. Performance Comparison

To assess the performance of all models, the average precision (AP) based on intersection-over-union (IoU) are introduced according to Pascal VOC Challenge. If a instance is predicted to the correct class, it is called true positive *TP*; if a instance is predicted as the other class, it is called false negative *FN*; if a instance is wrongly predicted to a class, with the mode considering the right classification, it is called false positive *FP*. Calculate the precision rate Per and recall rate Rec to achieve the AP evaluation

(2) $$Per=\frac{TP}{TP+FN},\;Rec=\frac{TP}{TP+FP}$$

The Average Precision is the area under the precision–recall curve of the detection task by averaging the precision over a set of spaced recall levels [0, 0.1, …, 1], and the mAP is the AP computed over all classes in our task.

(3) $$AP={\frac{1}{11}}_{rec\in \left\{0,0.1,\ldots ,1\right\}}p_{\mathrm{interp}}(Rec)$$

(4) $$p_{\mathrm{interp}}(Rec)=\max\underset{\tilde{r}ec:\tilde{r}ec\geq rec}{\max}Per(\tilde{R}ec)$$

where $Per(\tilde{R}ec)$ is the measure precision at recall $\tilde{R}ec$. Next, we compute the mAP averaged for an IoU = 0.5 (due to the complexity of the scenarios). The detection results are shown as follows Table 4. 

The comparison shows that, in our dataset, one-stage approach detection networks perform better than one-stage approach detection networks, such as the case of Faster R-CNN with VGG-16 with a mean AP of 83.53%, compared to the SSD with the same feature extractor VGG-16 that achieves 86.19% or RFB with the similar feature extractor VGG-19 with a mean AP of 85.23%. Another conclusion is that deep networks perform better than shallow networks. It is provided that a mean AP of YOLOv3 with deeper Darknet-53 achieving 91.44%, which are better than the mean AP 90.78% of YOLOv2 with lighter Darknet-19. In contrast, RetNet with ResNet50 performs at a mean AP of 92.79%, which is slightly better than other shallow models overall in the CropDeep datasets.

While Faster R-CNN has been a milestone of the two-stage detectors, these algorithms have some disadvantages in dealing with resources and time consumption for large datasets, which is not suitable to subsequent realistic application of object detection. SSD improves accuracy performance by producing different scale predictions and fusing feature maps of different layers. Similarly, RFB Net inspired by the receptive fields of human vision, proposes a novel RFB block module to significantly reduce the search space of objects. Combining the characteristics of the above models, YOLOv3 design a feature extractor with 53 convolutional layers (Darknet-53) to improve YOLOv2 in several aspects. Then feature pyramid networks and binary cross entropy loss were merged for class prediction in training. Furthermore, RetNet applied focus loss and bounding box regressor to reduce false positives resulting from class imbalance, which has been one of the best detectors with excellent performance in accuracy, speed, and complexity trade-offs.

Although the mean AP for the whole experiment shows a performance of more than 83% for the worst cases, some classes—such as lemon, spinach, scallion, and watermelon—show variable performance. Especially, the test results show that the accuracy of the all detection networks for lemon is relatively low. The reason is that the lemon has less training samples, and the lemon itself is a small target, which are difficult to current deep-learning detectors. After analysis, most of the lemons were misclassified into winter squash and unripe tomato, which have a similar color and shape. This illustrates that some classes with inter-class similarities in CropDeep are still challenging to recognition allowing for the complexity of the scenarios. In contrast, the accuracy of turnip is particularly low on the Faster R-CNN and RFB models. After analysis, Faster R-CNN and RFB do not extract the characteristics of turnip with relatively low classification confidence, which reduces the low accuracy of turnip detection. Furthermore, the number of samples is another fact that influences the generation of better results, since the implementation of deep learning systems requires a large number of data that can certainly influence the final performance. Using the deep-learning classification and detection network, the study shows several advantages of our CropDeep datasets when dealing with different crop species with various sizes, shapes, colors, etc., compared to previous works.

#### 5.2.2. Speed Evaluation

**Figure 8:**
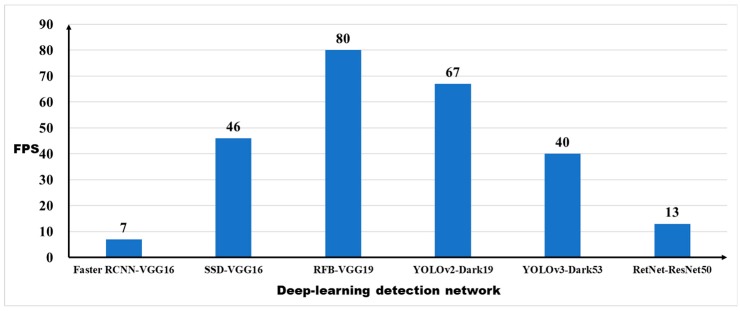
Speed performance of deep-learning detection networks.

Next, we explored the speed effect of the dataset on detection performance. Since the original intention of database collection is to construct the intelligent platform, which needs to be operated on various autonomous robots and equipment requiring not only accuracy, but also real-time performance, for further improving the overall timeliness and efficiency of precision agriculture management. Thus, the frames per second (FPS) was selected as the evaluation indicator to evaluate the speed performance of each detection networks on a same machine. The evaluation result of the detection time is shown in Figure 8.

The result shows that Faster R-CNN is too slow to achieve the effect of real-time detection and It can only reach 7FPS. SSD and YOLOv3 have the similar detection speed with approximate 40FPS, which can meet the basic needs for agricultural applications. YOLOv2 and RFB Net have the fastest detection model with detection speed over 67 FPS, especially RFB Net, with highest real-time detection speed up to 80 FPS, which is the best model for crop detection in terms of speed. However, RetNet with the best performance of detection accuracy just achieves a low speed performance with 13 FPS, which is unsuitable for real-time detection tasks in the CropDeep dataset.

#### 5.2.3. Loss Function

**Figure 9:**
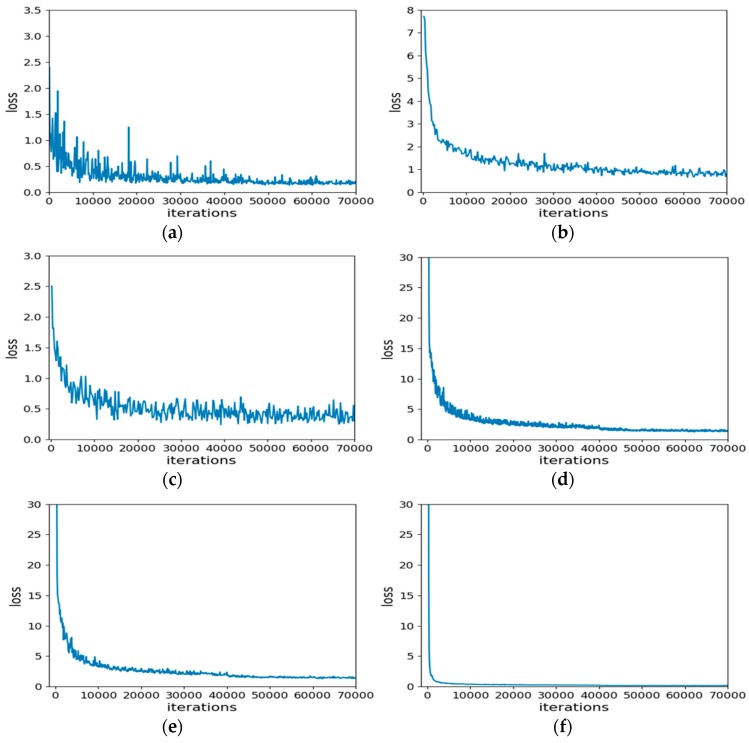
Loss function graphs of various detection networks. Left to right: (**a**) Faster RCNN loss, (**b**) SSD loss, (**c**) RFB loss, (**d**) YOLOv2 loss, (**e**) YOLOv3 loss, and (**f**) RetNet loss.

To estimate the prediction degree of each detection models, the loss function diagrams are drawn as follows in Figure 9.

**Figure 10:**
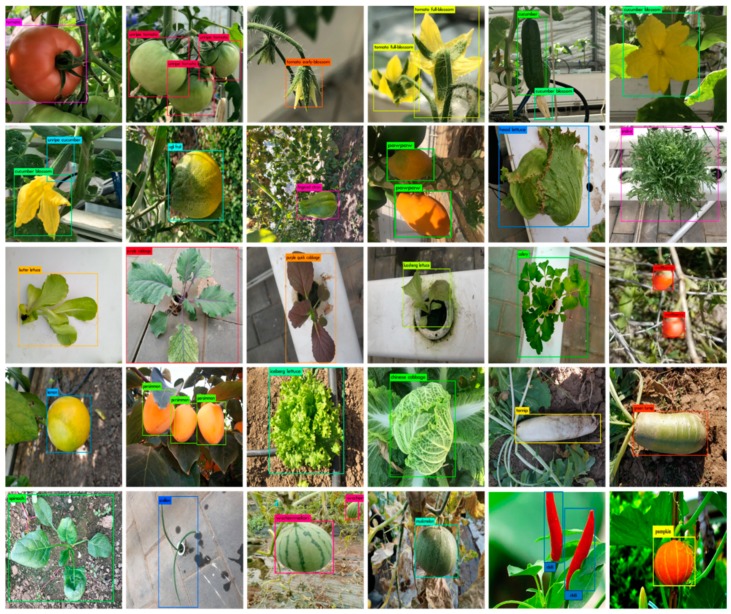
Sample detection results for 30 crop class. We see that small and similar objects pose a challenge for classification, even when localized well, in our CropDeep dataset.

The comparative results of loss function show that the trend of the loss function of all detection meta-architectures is reduced. It stabilizes at about 50,000 Iterations, which the predicted value of the network was closer to the true value. Among them, the loss function of Faster R-CNN, SSD, and RFB has large fluctuations in the process of descent, while the loss function of YOLOv2 and YOLOv3 is relatively smooth. In comparison, the loss function of RetNet decreases faster and smoother, which indicates the learning ability of the network is stronger. Finally, with comprehensive consideration of speed, accuracy and robustness, we suggest that the YOLOv3 network has plenty of room for improvements on end-to-end fine-grained detection tasks in greenhouse. It is still vital and necessary to develop more advanced models for crop detection in our CropDeep dataset. While the RetNet obtained the highest overall performance on our CropDeep dataset, which has the potential for further improvement by reducing detection time and the GPU memory consumption. The sampled detection results of each class offered by RetNet are present in Figure 10. From left to right and top to bottom, the crop classes are respectively tomato, unripe tomato, tomato early-blossom, tomato full-blossom, cucumber, cucumber blossom, unripe cucumber, winter squash, fingered citron, pawpaw, head lettuce, endive, butter lettuce, rutabaga, purple cabbage, Luosheng lettuce, celery, wolfberry, lemon, persimmon, iceberg lettuce, Chinese cabbage, turnip, green turnip, spinach, scallion, watermelon, muskmelon, chili pepper, and pumpkin.

**Figure 11:**
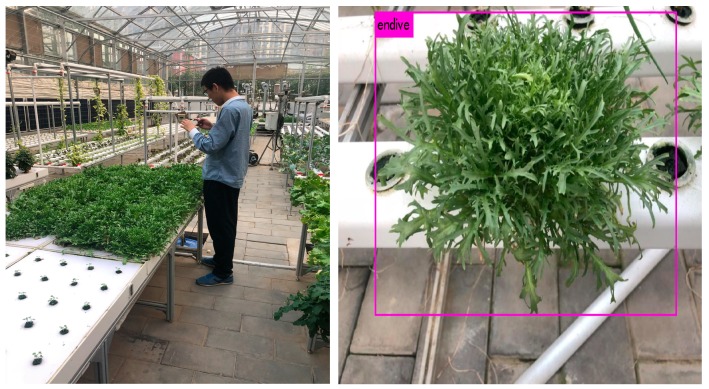
Test results of practical application in agricultural greenhouse.

The CropDeep allows us to study online and offline agricultural applications in a real-world environment. IOT cameras and mobile cameras can accurately take photos of crops in greenhouses to capture accurate photos of crop growth. The captured images provide input to the system and train the network model. The deep learning model can improve the accuracy of crop detection and classification and can achieve real-time detection speed. The results of the model analysis effectively evaluate the growth status of the crop. Crop managers manage greenhouse greenhouses through the results of tests on smartphones to achieve precise agricultural management. The test results of our system are shown in Figure 11.

## 6. Conclusions

In this work, we present a domain-specific vision dataset of crops, namely CropDeep, in the field of precision agriculture. The novelty of CropDeep is that it aims at providing the data benchmark to constructing deep-learning-based classification and detection models according to realistic characteristics of agricultural tasks in greenhouses. In contrast to many existing vision datasets, the advantages of CropDeep include: (1) the datasets are collected by various cameras and equipment in greenhouses, which are more useful in offering management services for precision agriculture than previous datasets. (2) The annotation samples of each crop class are more representative and abundant than previous works, which support a stronger benchmark for technological applications of deep-learning-based models in agricultural tasks. (3) The images of the datasets contain periodic changes and similarity characteristics, which represent a long-tail real-world challenge in classification and detection problems.

Agricultural picking robots need to identify and classify crops. In order to improve the accuracy of crop detection, we need to use a large number of crop pictures for training. CropDeep, in contrast to many existing computer datasets: (1) is unbiased because it was collected by non-computer vision researchers for a clear purpose; (2) includes 31 crops in common greenhouses, (3) has high similarity among some categories in the dataset; and (4) contains the state of the entire growth cycle of some crops.

The introduction of CropDeep enables us to study online and offline agricultural applications in a realistic world setting. To verify that the effective application of CropDeep on classification and detection, we ran experiments with several deep-learning-based frameworks utilizing different species. While our baseline classification and detection results are encouraging, state-of-the-art deep-learning models have room to improve when applied to crops production and management. Finally, with comprehensive consideration of speed, accuracy, and robustness, we suggest that the YOLOv3 network has plenty of room for improvements on end-to-end fine grained detection tasks in greenhouse.

In the future, we plan to add more images and annotations of new crop species for fine-gained classes that were challenging to annotate. We also plan to explore to improve the performance of deep-learning classification and detection frameworks for more agricultural applications. Finally, we expect this dataset to be useful in studying how to teach fine-grained visual categories to humans.

## Figures and Tables

**Table 1:** Summary of popular general and fine-grained vision datasets with plants.

Dataset	Classes	Image Number	Annotation Samples Number
Flowers 102	102	1020	0
CUB 200-2011	200	5994	0
Urban Trees	18	14,572	0
LeafSnap	185	30,866	0
ImageNet	1000	14,197,122	1,034,908
MS-COCO	80	300,000	More than 2,000,000
AI Challenge	61	47,393	0
PlantVillage	38	19,298	0
iNat2017	5089	858,184	561,767
VegFru	70	160,731	0
CropDeep	31	31,147	49,765

**Table 2:** Images and annotated samples number of each category in CropDeep dataset.

No.	Categories	Images Number	Annotated Samples Number	Annotated Percentage (%)
1	Tomato (*Lycopersicon esculentum Mill.*)	1021	1543	3.10
2	Unripe tomato (*Lycopersicon esculentum Mill.*)	898	1367	2.75
3	Tomato early-blossom (*Lycopersicon esculentum Mill.*)	985	1914	3.85
4	Tomato full-blossom (*Lycopersicon esculentum Mill.*)	1083	1820	3.66
5	Cucumber (*Cucumis Linn.*)	972	1287	2.59
6	Cucumber blossom (*Cucumis Linn.*)	1112	1646	3.31
7	Unripe cucumber (*Cucumis Linn.*)	898	1382	2.78
8	Winter squash (*Cucurbita maxima Duch.*)	971	1429	2.87
9	Fingered citron (*Sechium edule Jacq.*)	1083	1588	3.19
10	Pawpaw (*Chaenomeles Lindl.*)	930	1704	3.42
11	Head lettuce (*Lactuca sativa Linn.*)	916	1373	2.76
12	Endive (*Picris divaricata Vaniot.*)	951	1785	3.59
13	Butter lettuce (*Lactuca sativa Linn.*)	908	1527	3.07
14	Rutabaga (*Brassica napobrassica Mill.*)	1116	1764	3.54
15	Purple cabbage (*Brassica oleracea Linn.*)	977	1379	2.77
16	Luosheng lettuce (*Lactuca sativa Linn.*)	1294	1840	3.70
17	Celery (*Apium graveliens Linn.*)	1047	1739	3.49
18	Wolfberry (*Lycium barbarum Linn.*)	952	1421	2.86
19	Lemon (*Citrus limon Burm.*)	1113	1545	3.10
20	Persimmon (*Diospyros kaki Thunb.*)	1099	1893	3.80
21	Iceberg lettuce (*Lactuca sativa Linn.*)	923	1802	3.62
22	Chinese cabbage (*Brassica oleracea Linn.*)	1094	1594	3.20
23	Turnip (*Raphanus sativus Linn.*)	1029	1629	3.27
24	Green turnip (*Raphanus sativus Linn.*)	951	1903	3.82
25	Spinach (*Spinacia oleracea Linn.*)	1057	1557	3.13
26	Scallion (*Allium fistulosum Linn.*)	1033	1647	3.31
27	Watermelon (*Citrullus lanatus Matsum.*)	960	1573	3.16
28	Muskmelon (*Cucumis melo Linn.*)	1190	1896	3.81
29	Chili pepper (*Capsicum annuum Linn.*)	1026	1821	3.66
30	Pumpkin (*Cucurbita moschata Duch.*)	983	1652	3.32
31	Person	575	745	1.50
	Total	31147	49765	100

**Table 3:** Averaging accuracy across all species computed by seven classification models.

Categories	VGG16	VGG19	SqueezeNet	InceptionV4	DenseNet121	ResNet18	ResNet50
Tomato	100	100	97.9	81.63	100	100	100
Unripe tomato	97.9	100	97.9	100	100	100	100
Tomato early-blossom	96.5	96.3	90.9	85.1	98.4	98.1	98.8
Tomato full-blossom	97.5	97.2	92.3	90.2	100	100	100
Cucumber	96.2	98.1	90.4	88.5	98.08	96.2	98.1
Cucumber blossom	96.9	97.1	88	86.3	98.5	96.7	98.7
Unripe cucumber	100	100	93.5	100	100	100	100
Winter squash	95.2	100	87.3	100	100	100	100
Fingered citron	100	100	98.1	100	100	100	100
Pawpaw	100	100	100	100	100	100	100
Head lettuce	97.3	98.3	96.5	100	98.8	100	100
Endive	100	100	95.9	100	100	100	100
Butter lettuce	100	100	100	94.8	100	100	100
Rutabaga	100	98.2	89.6	100	100	100	100
Purple cabbage	100	100	89.5	100	100	100	100
Luosheng lettuce	98.2	100	92.6	100	100	100	100
Celery	100	98.5	92.5	100	100	100	100
Wolfberry	100	97.7	97.7	90.7	97.6	100	100
Lemon	100	100	95.5	100	100	100	100
Persimmon	98.77	100	98.8	98.7	100	100	100
Iceberg lettuce	100	100	100	100	100	100	100
Chinese cabbage	100	100	100	100	100	100	100
Turnip	100	100	89.7	100	100	100	100
Green turnip	100	98.8	91.6	100	98.8	100	100
Spinach	100	100	94	100	100	100	100
Scallion	100	100	95.7	96.7	100	100	100
Watermelon	100	100	90.3	100	100	100	100
Muskmelon	97.7	97.8	84.2	100	100	100	100
Chili pepper	98.2	97.5	87.1	97.9	100	100	100
Pumpkin	96.3	96.3	85.1	97.2	100	100	100
Person	88.8	92.3	81.6	96.1	96.3	97.3	98.6
Average accuracy	98.56	98.84	93.03	96.89	99.56	99.62	99.81

**Table 4:** Detection results offered by different models.

Detection Architectures	Faster R-CNN	SSD	RFB	YOLOv2	YOLOv3	RetNet
Feature Extractor	VGG-16	VGG-16	VGG-19	Darknet-19	Darknet-53	ResNet50
Tomato	90.82	90.91	90.45	97.28	97.51	98.82
Unripe tomato	88.76	89.18	90.18	93.3	92.34	97.62
Tomato early-blossom	84.16	79.82	85.17	83.02	88.42	91.09
Tomato full-blossom	90.13	87.11	89.79	91.28	88.41	85.1
Cucumber	79.87	80.34	80.37	88.41	86.85	83.21
Cucumber blossom	76.12	75.74	84.29	81.85	81.07	84.09
Unripe cucumber	75.47	82.05	86.17	88.01	80.5	85.21
Winter squash	91.48	90.91	90.8	96.18	96.06	92.73
Fingered citron	94.6	99.18	95.19	99.38	98.29	100
Pawpaw	67.17	78.89	81.38	97.52	97.36	98.61
Head lettuce	95.76	97.02	95.47	98.79	96.21	99.38
Endive	89.02	87.92	89.12	91.81	88.78	87.54
Butter lettuce	88.43	90.46	90.57	96.03	96.79	99.15
Rutabaga	87.71	88.08	88.18	97.42	98.56	100
Purple cabbage	88.79	89.15	87.99	95.3	94.47	97.61
Luosheng lettuce	95.29	100	100	100	97.71	99.3
Celery	75.74	88.24	81.15	76.62	82.67	87.61
Wolfberry	90.33	98.42	99.86	96.81	99.35	98.11
Lemon	55.44	60.54	52.96	63.94	70.39	74.28
Persimmon	90.46	80.22	89.09	82.54	85.65	89.63
Iceberg lettuce	95.65	90.91	90.91	98.61	99.14	100
Chinese cabbage	100	100	100	100	100	100
Turnip	43.53	80.37	21.76	100	100	100
Green turnip	91.31	96.97	90.7	98.91	99.97	100
Spinach	63.64	63.64	63.64	62.12	63.6	66.25
Scallion	73.02	72.73	81.06	76.42	82.54	85.62
Watermelon	80.01	79.81	84.5	90.41	89.12	91.33
Muskmelon	90.17	86.58	90.62	86.78	88.93	87.36
Chili pepper	88.73	87.62	88.41	94.92	100	100
Pumpkin	83.26	88.27	89.71	92.9	96.15	97.54
Person	84.71	90.91	92.62	97.63	97.88	99.3
Average mAP	83.53	86.19	85.23	90.78	91.44	92.79
